# Radiation myelitis after durvalumab administration following chemoradiotherapy for locally advanced non-small cell lung cancer: an illustrative case report and review of the literature

**DOI:** 10.1007/s13691-019-00367-5

**Published:** 2019-03-13

**Authors:** Katsumaro Kubo, Koichi Wadasaki, Hiroaki Yamane, Mihoko Doi

**Affiliations:** 1 Department of Radiation Oncology, Hiroshima Prefectural Hospital, 1-5-54 Ujinakanda Minami-ku Hiroshima-shi, Hiroshima, 734-0004 Japan; 2 Department of Clinical Oncology, Hiroshima Prefectural Hospital, 1-5-54 Ujinakanda Minami-ku Hiroshima-shi, Hiroshima, 734-0004 Japan

**Keywords:** Chemoradytherapy, Radiation myelitis, Durvalumab

## Abstract

A 69-year-old man with stage IIIB lung adenocarcinoma received durvalumab following chemoradiotherapy. The prescribed dose was 50 Gy in 2 Gy fractions, and the maximum spinal cord dose was 40 Gy. After three cycles of durvalumab, he experienced bladder and rectal disturbance, muscle weakness in the lower limbs, and sensory loss in the lower body. Magnetic resonance imaging revealed T2 signal hyperintensity involving the thoracic spinal cord. As the thoracic spinal cord with T2 signal hyperintensity matched with the irradiated site, the patient was diagnosed with radiation myelitis. This case report shows the clinical and radiographic features of a case of locally advanced non-small cell lung cancer that demonstrated radiation myelitis following durvalumab administration. The time of onset was very early and the influence of durvalumab was suspected as the cause of myelitis.

## Introduction

Immunotherapy has become an important biological therapy in lung cancer treatment. Durvalumab is a selective, high-affinity, human IgG1 monoclonal antibody that blocks programmed death-ligand 1 (PD-L1) from binding to programmed death 1 and CD80, allowing T cells to recognize and eliminate tumor cells [1–3]. The PACIFIC study showed that durvalumab as a consolidation therapy improved the overall survival compared with the placebo therapy in patients with non-small cell lung cancer (NSCLC) treated with definitive chemoradiotherapy (CRT) [4].

This result had a great impact worldwide, and durvalumab as consolidation therapy following CRT became a new standard treatment. In Japan, durvalumab was approved as a consolidation therapy after definitive CRT in locally advanced NSCLC in July 2018. Consolidation therapy using durvalumab is predicted to increase in the future. However, as a new treatment, there are insufficient data that support on its adverse events. Additionally, there is no report on myelitis after durvalumab administration following CRT.

In this report, we present the clinical and radiographic features of a case of locally advanced NSCLC treated with CRT that demonstrated radiation myelitis following durvalumab administration as a consolidation therapy.

## Case report

The patient was a 69-year-old man with stage IIIB lung adenocarcinoma (T1bN3M0, Union for International Cancer Control 8th edition) with primary tumor in the left lower lobe and multiple lymph node metastases (#2R, 4R, 7 and 10L). He had no past medical history and complications. Both epidermal growth factor receptor mutations and anaplastic lymphoma kinase status were negative. The PD-L1 expression was 0%. He had received chemotherapy as an initial treatment because CRT was not suitable for the wide extent and size of the tumor. Four cycles of carboplatin, pemetrexed, and bevacizumab were administered. Subsequently, he was treated with pemetrexed and bevacizumab as maintenance therapy. Although the tumor decreased in size once, it regrew. As the tumor lesions were still large, but the tumor size was smaller than that before initial treatment, we judged that CRT was possible at this timing. Therefore, we decided to perform CRT followed by durvalumab as the next treatment on the cancer board. CRT was performed 1 month after the last administration of pemetrexed and bevacizumab. For the large tumor lesions, we prescribed up to 50 Gy in 2 Gy fractions with involved-field radiotherapy to avoid the occurrence of adverse effects. The dose was given through the parallel-opposed anteroposterior portals up to 30 Gy, and multiportal beams were used to reduce the dose in the spinal cord from 30 to 50 Gy. Weekly carboplatin (area under the curve, 2.0) and paclitaxel (40 mg/m^2^) were administered concomitantly. No major adverse events were observed during CRT. One month after CRT, the tumor decreased in size and there was no new lesion or adverse effect, such as esophagitis and pneumonitis. Therefore, durvalumab (10 mg/m^2^, every 2 weeks) was started.

**Fig. 1:**
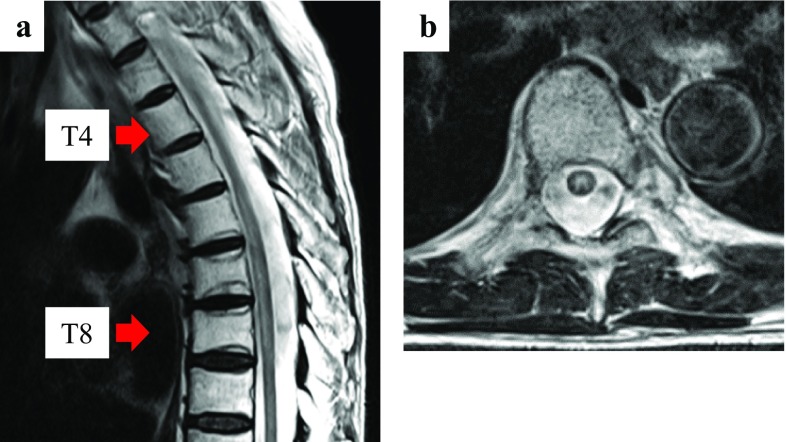
Magnetic resonance imaging 2.5 months after the completion of CRT. **a** Sagittal: T2 signal hyperintensity lesion between T5 and T8 spinal levels. **b** Axial: T2 signal hyperintensity lesion in the center of the spinal cord at T7 spinal level

**Fig. 2:**
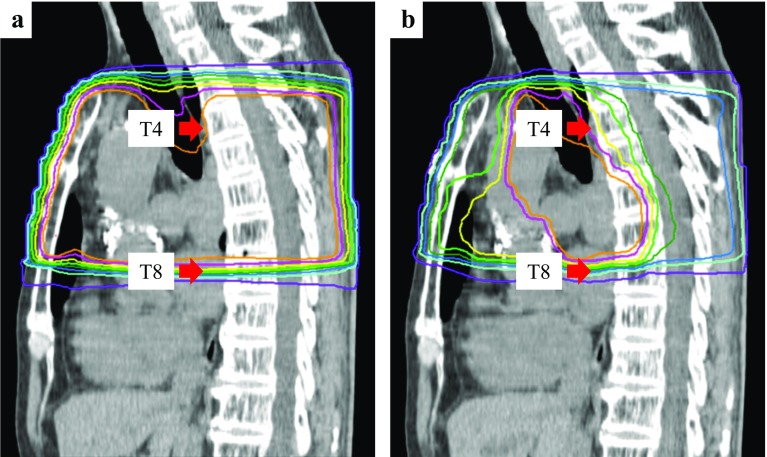
Dose distribution of the plan up to 30 Gy (**a**) and from 30 to 50 Gy (**b**). Isodose lines from outer to inner represent 20%, 40%, 50%, 60%, 70%, 80%, 90%, and 95%, respectively, of the prescribed dose. The thoracic spinal code (T4–T8 levels) was irradiated. The thoracic spinal cord was covered at 95% line of the prescribed dose up to 30 Gy and at 50% line from 30 to 50 Gy

**Fig. 3:**
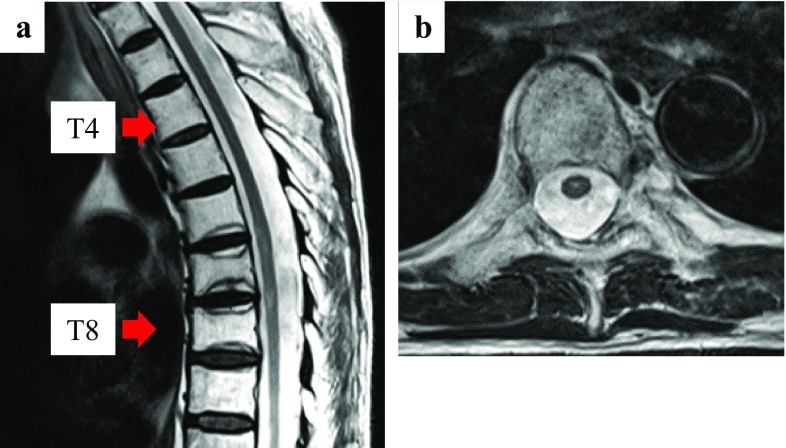
Magnetic resonance imaging 1 week after the starting date of steroid treatment. **a** Sagittal: T2 signal hyperintensity lesion between T5 and T8 spinal levels was unclear. **b** Axial: T2 signal hyperintensity lesion in the center of the spinal cord at T7 spinal level shrunk

After three courses of durvalumab (2.5 months after the completion of CRT), the patient experienced dysuria. The next day, he experienced muscle weakness of the lower limbs and difficulty in walking independently. Physical examination revealed bladder and rectal disturbance, muscle weakness in the lower limbs, and sensory loss in the lower body. Magnetic resonance imaging (MRI) was performed and revealed T2 signal hyperintensity involving the thoracic spinal cord (T5–T8 levels) (Fig. 1). The thoracic spinal cord with T2 signal hyperintensity matched the irradiated site. The doses to the thoracic spinal cord were 30 Gy in 2 Gy fractions up to 30 Gy and 10 Gy in 1 Gy fraction from 30 to 50 Gy (Fig. 2). Thus, the total dose was 40 Gy. There were no findings of infection and tumor cells in the cerebrospinal fluid. As there was no evidence of other causes, such as epidural metastasis or spinal cord compression secondary to vertebral metastases, and as T2 signal abnormalities matched the irradiated site of the thoracic spinal cord, we diagnosed radiation myelitis accompanying treatment for NSCLC. We stopped durvalumab administration and started steroid treatment. The patient received intravenous methylprednisolone pulses of 1.0 g/day for three consecutive days, followed by oral prednisolone at an initial dosage of 50 mg/day. The oral prednisolone was gradually tapered by 5 mg every 1 week. One week after the starting date of steroid treatment, muscle weakness in the lower limbs gradually improved and crutch walking independently became possible, though bladder and rectal disturbance remained. The follow-up MRI (1 week after onset) revealed significant improvement of the T2 signal abnormalities (Fig. 3).

## Discussion

Though radiation-induced spinal cord injury is rare, it can be severe, resulting in pain, paresthesias, sensory deficits, paralysis, Brown-Sequard syndrome, and bladder and rectal disturbance. The initial symptoms are usually paresthesias and sensory changes that start 9–15 months after the completion of radiation therapy [5]. MRI is helpful in establishing the diagnosis and may reveal spinal cord edema or atrophy, T1-weighted hypointensity, or T2-weighted hyperintensity. Using conventional fractionation, the probability of myelopathy was reported to be 0.03% at 45 Gy and 0.2% at 50 Gy, respectively [5, 6]. Though concomitant intravenous use of chemotherapy including cisplatin can enhance neurotoxicity [7], total dose of less than 50 Gy rarely causes radiation myelitis. In our case, the dose to the thoracic spinal cord (T4–T8 levels) was 30 Gy in 15 fractions plus 10 Gy in 10 fractions. According to previous reports, the total irradiated dose to the thoracic spinal cord was 36.5 Gy, converted into 2 Gy/fraction equivalents with *α*/*β* as 0.87. Moreover, in this case, the onset of radiation myelitis was 2.5 months after the completion of CRT and was very early, compared with the previous reports. This case was very unique in that the onset of radiation myelitis was very early and the low-dose caused radiation myelitis. Accordingly, it was suspected that the factor except radiation influenced myelitis.

Our patient received chemotherapy, including bevacizumab, as the first treatment. Bevacizumab is a humanized, antivascular endothelial growth factor monoclonal IgG1 antibody. Bevacizumab might cause the onset of radiation myelitis due to ischemia or infarction. However, there is no report of myelitis following carboplatin, pemetrexed, and bevacizumab administration, and the onset of myelitis was deviated from the administration of bevacizumab. Weekly carboplatin and paclitaxel, which are standard regimens, were administered during CRT. Though concurrent chemotherapy can enhance neurotoxicity, there is no report stating that the low-dose radiation therapy with carboplatin and paclitaxel resulted in the onset of radiation myelitis.

Neurological adverse events associated with immune checkpoint inhibitors (ICIs) less commonly include drug toxicities [8]. The influence of ICIs on neurological adverse events by radiation therapy is unknown. Tumor necrosis factor (TNF) is produced in the central nervous system by microglia. TNF is involved in nerve degeneration and inflammation, and radiation therapy enhances TNF production by microglia [9, 10]. Since PD-L1 is also expressed in the central nerve, it would be considered that PD-L1 was highly expressed under the inflammation of CRT or TNF production, and immune tolerance was induced. In this case, we assumed that the immune tolerance might be inhibited by durvarumab and the inflammation was enhanced. Therefore, myelitis developed. In addition, a report has shown the association between immunotherapy after radiotherapy and radiation necrosis [11]. Thus, ICIs might have some influence on the irradiated central nervous system. Therefore, it would be reasonable to consider that in this case, radiation myelitis was caused by the influence of durvalumab on CRT.

The treatment of immunotherapy-related neurological adverse events has been reported [8]. If the adverse event is considered severe, administration of a high dose of systemic corticosteroids can be recommended. Treatment with intravenous immunoglobulin and plasmapheresis could be considered. Our patient discontinued durvalumab administration and received steroid treatment. Though the symptoms have not completely improved, the follow-up MRI showed significant improvement of the T2 signal abnormalities and steroid treatment seemed to be effective.

This case report shows the clinical and radiographic features of a case of a locally advanced NSCLC that demonstrated radiation myelitis after durvalumab administration following CRT. As durvalumab administration following CRT is a new treatment strategy and there is a possibility that pathological conditions, same with the present case report, might occur in the future, we reported this case to call everybody’s attention and encourage careful observation.
